# Does diabetes affect paraneoplastic thrombocytosis in colorectal cancer?

**DOI:** 10.1515/med-2021-0407

**Published:** 2022-01-13

**Authors:** Gyorgy Herczeg, Aniko Somogyi, Magdolna Herold, Agnes Fodor, Klara Rosta, Magdolna Dank, Zsolt Lang, Zoltan Herold

**Affiliations:** Department of General Surgery, Szent Imre University Teaching Hospital, Budapest, Hungary; Department of Internal Medicine and Haematology, Semmelweis University, Budapest, Hungary; Department of Obstetrics and Gynecology, Medical University of Vienna, Vienna, Austria; Department of Internal Medicine and Oncology, Division of Oncology, Semmelweis University, Budapest, Hungary; Department of Biomathematics and Informatics, University of Veterinary Medicine Budapest, Budapest, Hungary; Department of Internal Medicine and Haematology, Semmelweis University, Szentkiralyi utca 46., H-1088 Budapest, Hungary; Department of Internal Medicine and Oncology, Division of Oncology, Semmelweis University, Tomo utca 25-29., H-1083 Budapest, Hungary

**Keywords:** colorectal neoplasms, diabetes mellitus, type 2, interleukin-6, thrombocytosis, thrombopoietin

## Abstract

**Background:**

A large variety of factors can affect colorectal cancer (CRC) survival, including type 2 diabetes mellitus (T2DM) and paraneoplastic thrombocytosis. Although several common factors play a role in their development and platelets are damaged in both diseases, the combined relationship of the three conditions was never investigated previously.

**Methods:**

A prospective, real-life observational cohort study was conducted with the inclusion of 108 CRC patients and 166 voluntary non-CRC subjects. Plasma interleukin-6 and thrombopoietin levels were measured.

**Results:**

Study participants were divided into cohorts based on the presence of T2DM. Platelet count (*p* < 0.0500) and interleukin-6 (*p* < 0.0100) level were significantly higher in the CRC groups. Thrombopoietin level was higher in the T2DM, CRC, and *CRC + T2DM* groups (*p* < 0.0500). Analysis of parameter changes over time and survival models revealed that neither platelet count, interleukin-6, nor thrombopoietin levels were affected by T2DM. Death of patients was associated with higher baseline platelet count (*p* = 0.0042) and interleukin-6 level (*p* < 0.0001).

**Conclusion:**

Although the independent, disease-worsening effect of paraneoplastic thrombocytosis and T2DM is known, the coexistence of the two did not further impair the survival of CRC patients, suggesting that T2DM has no significant effect over paraneoplastic thrombocytosis.

## 1 Introduction

Thrombocytosis – platelet counts above the upper value of normal range (usually ≥400 × 10^9^/L) – is described as a poor prognostic sign in colorectal cancer (CRC). Both preoperative and postoperative thrombocytoses are associated with worse patient survival [1,2]. Thrombocytosis may develop for several reasons, such as the bleeding of the tumor or a metabolic change caused by the tumor itself called paraneoplastic thrombocytosis [1,3]. The proposed paracrine-signaling pathway of paraneoplastic thrombocytosis [3,4] includes the overproduction of various cytokines (e.g., interleukin-6) by the tumor, which causes increased hepatic thrombopoietin production that modulates the production of platelets within the bone marrow, ultimately resulting in an increased platelet count.

Diabetes mellitus is one of the most prevalent diseases in our time; based on the latest estimations available in the ninth edition of IDF Diabetes Atlas [5], the incidence of the disease varies between 4 and 10.4%, with over 460 million diabetes patients around the world [5]. Approximately 90% of diabetes patients are suffering from type 2 diabetes mellitus (T2DM), which develops in later ages [6], similar to that of CRC [7]. Over the age of 60 years, T2DM may occur in every fourth or fifth person [5,8], and compared to the healthy population, an increased occurrence of malignancies is confirmed in T2DM [9,10,11]. CRC occurs approximately 1.5× more often in patients with T2DM [12,13]; furthermore, the coexistence of T2DM is associated with an increased risk for a shorter survival in CRC [14,15]. Similar to CRC, T2DM can be also described by various platelet abnormalities and an increased thrombopoietin production is also known [16,17,18].

Although several potential mechanisms link cancers and T2DM together [19], and platelets are affected in both diseases [1,2,3,4,16,17,18], the relationship among T2DM, (paraneoplastic) thrombocytosis, and CRC is poorly investigated. An earlier retrospective study [20] from our group has found that a personalized thrombocytosis measure calculated from preoperative and precancer platelet counts can better predict the patient survival, and it has been associated with various clinicopathological parameters, including T2DM. Although our earlier study has revealed some possible connections between the three conditions, the exact biological effect of T2DM on CRC-related paraneoplastic thrombocytosis could not have been investigated via that retrospective manner. Therefore, a prospective, real-life observational cohort study was conducted where paraneoplastic thrombocytosis was investigated within diabetic and nondiabetic CRC cohorts through the changes in platelet counts, plasma interleukin-6, and thrombopoietin levels.

## 2 Methods

### 2.1 Patients and study design

A prospective, real-life observational cohort study was carried out. A total of 108 patients diagnosed with CRC and 166 voluntary non-CRC subjects were enrolled for the study between 2017 and 2019. CRC patients attended at both the Department of Internal Medicine and Hematology, Semmelweis University, Budapest and the Department of General Surgery, Szent Imre University Teaching Hospital, Budapest. Written informed consent was collected from all study participants before performing any study-specific procedures. Exclusion criteria included age <18 years, any previous malignancies, known inflammatory bowel- and/or chronic kidney- and/or systemic autoimmune- and/or inadequately controlled thyroid- and/or hematologic- and/or any mental diseases, the usage of erythropoiesis-stimulating agents and/or recent blood transfusion, and patients with an Eastern Cooperative Oncology Group (ECOG) performance status >2.

Voluntary non-CRC subjects consisted of healthy young controls, older controls, and T2DM patients. Rationale of the inclusion of healthy young subjects was that some of the measured parameters have no reference values and that the older volunteers included both completely healthy individuals and nonmetabolic disease patients (e.g., hypertension). T2DM patients attended at the Metabolic Outpatient Clinic of the Department of Internal Medicine and Hematology, Semmelweis University, Budapest. In addition to the exclusion criteria described by CRC patients above, voluntary nondiseased subjects were excluded in the presence of prediabetes or any other metabolic disorders.

### 2.2 Clinical and laboratory data measurements

Anamnestic data including comorbidities and recent medications, body weight, and height were collected, and fasting blood samples were drawn according to the following protocol: (1) at the diagnosis of CRC, before any oncological treatments or the surgical resection of the primary tumor, (2) at least 6 weeks, (3) 6 months, and (4) 12 months after the tumor removal surgery. At the time of postoperative measurements, oncological treatments were stopped/paused only as part of the patients’ standard of oncological care, and sampling was performed only at least 6 weeks after the last treatment due to the known platelet influencing effects of several cytotoxic regimens [21,22]. If it was not feasible to stop/pause the oncological treatment, in the best interest of the patient, the visit was omitted. Complete blood count, aspartate- and alanine transaminase, gamma-glutamyl transferase, plasma glucose, and creatinine were measured at the Central Laboratory of Semmelweis University and at the Central Laboratory of Szent Imre University Teaching Hospital. Estimated glomerular filtration rate was calculated using the Chronic Kidney Disease-Epidemiology Collaboration equations [23]. Plasma interleukin-6 and thrombopoietin levels were measured using the ELECSYS^®^ Interleukin-6 electrochemiluminescence immunoassay (ECLIA) (Roche Diagnostics GmbH, Mannheim, Germany) and the Human Thrombopoietin Quantikine^®^ enzyme-linked immunosorbent assay (ELISA) (catalog number: DTP00B, R&D Systems, Minneapolis, MN, USA) kits, respectively. Plasma thrombopoietin level was measured from platelet-poor plasma, as per manufacturer description.

Side of CRC was described as right sided if the tumor was originating from cecum, ascending colon, and proximal two-third of the transverse colon and left sided if originating from the distal one-third of the transverse colon, descending colon, sigmoid colon, and rectum [24]. Staging was given by histopathological examination of surgical specimens and imaging studies; the American Joint Committee on Cancer grouping was used [25]. The usage of biological agents was recorded as a dummy variable and chemotherapy was grouped as adjuvant if no metastasis and first-line, second-line, etc., if metastasis was present. The overall survival of patients was defined as the length of time from the date of CRC diagnosis until death. Follow-up of cancer patients was terminated on January 31, 2021, patients alive at this time point were right censored.

### 2.3 Statistical analysis

Statistical analysis was performed with R version 4.0.5 [26]. Wilcoxon–Mann–Whitney *U* test, Fisher’s exact test, Kruskal–Wallis test with *p*-value corrected pairwise Wilcoxon–Mann–Whitney *U* tests as post-hoc, and Spearman rank correlation were used. To detect the changes of various parameters in time, linear mixed effect models were used (R-package *nlme* [27]). Survival of patients was analyzed for both preoperative and longitudinal data with Cox regression models and Bayesian univariate and multivariate joint survival models (R-package *rstanarm* [28]), respectively. *p* < 0.05 was considered as statistically significant, and *p*-values were corrected with the Holm method [29] for the problem of multiple comparisons. Compared to the “conventional” frequentist methods, Bayesian methods give only a probability distribution for the investigated parameter but no *p*-values. Interpretation of joint model results was as follows. If the Bayesian equivalent to the frequentist confidence interval (CI), the 95% credible interval contained the hazard ratio (HR) of 1, the model was considered as clinically not relevant. However, if the 95% credible interval was less than or more than HR = 1, the effect of the parameter was considered as a good or bad sign of patient survival, respectively. Results were expressed as mean ± standard deviation and as the number of observations (percentage) for continuous and count data, respectively. Naïve Kaplan–Meier and longitudinal survival curves were drawn with the R-package *survminer* (Kassambara, Kosinski, and Biecek, version 0.4.9, 2021) and the built-in methods of *rstanarm*, respectively.


**Informed consent:** Informed consent has been obtained from all individuals included in this study.
**Ethical approval:** The study was approved by the Regional and Institutional Committee of Science and Research Ethics, Semmelweis University (SE TUKEB 21-12/1994, approval date of latest modification: February 10, 2017), by the Institutional Review Board of Szent Imre University Teaching Hospital (SZIK IKEB 5/2017), and by the Committee of Science and Research Ethics, Hungarian Medical Research Council (ETT TUKEB 8951-3/2015/EKU).

## 3 Results

A total of 274 study participants were included: 108 CRC patients and 166 voluntary, non-CRC subjects. CRC patients were divided into two cohorts based on the presence of T2DM. Patients without diabetes (*n* = 82) were assigned to the “*CRC*” group, whereas patients with a history of T2DM (*n* = 26) were assigned to the “*CRC* + *T2DM*” group. Duration of diabetes was 6.88 ± 6.10 years, from which T2DM was diagnosed in four cases around the time of CRC diagnosis.

In most parameters, the two tumor groups did not differ from each other, whereas plasma glucose was significantly higher (*p* = 0.0018), and tendentiously higher white blood cell-, neutrophil-, eosinophil-, and monocyte counts were observed in the *CRC + T2DM* group. Platelet aggregation inhibition therapy was more common in *CRC + T2DM* patients; no difference was found in radiotherapy (neoadjuvant only), whereas no-line or late-line chemotherapy was more common in those CRC patients with T2DM than those of without (*p* = 0.0560; Table 1).

**Table 1:** Metabolic, clinical, and other parameters of CRC patients

Parameter	CRC	*CRC + T2DM*	*p*-value
	(*n* = 82)	(*n* = 26)	
Age (years)	67.42 ± 9.33	70.83 ± 7.24	0.3919
Sex (male:female)	51:31 (62.2:37.8%)	20:6 (76.9:23.1%)	0.3778
Body mass index (kg/m^2^)	27.26 ± 3.87	27.43 ± 4.64	0.7778
Systolic blood pressure (mmHg)	144.49 ± 18.48	141.12 ± 19.55	0.7654
Diastolic blood pressure (mmHg)	78.99 ± 10.19	78.20 ± 9.10	0.7527
Heart rate (1/min)	77.88 ± 13.76	80.76 ± 16.36	0.7654
White blood cell count (10^9^/L)	8.14 ± 2.91	11.11 ± 7.46	0.3919^*^
Neutrophil count (10^9^/L)	5.60 ± 2.57	7.87 ± 5.59	0.3919^*^
Eosinophil count (10^9^/L)	0.18 ± 0.16	0.59 ± 1.73	0.3919^*^
Basophil count (10^9^/L)	0.06 ± 0.05	0.06 ± 0.04	0.7654
Monocyte count (10^9^/L)	0.61 ± 0.46	0.78 ± 0.51	0.3919^*^
Lymphocyte count (10^9^/L)	1.74 ± 1.11	1.81 ± 0.92	0.7654
Red blood cell count (10^12^/L)	4.50 ± 0.58	4.42 ± 0.52	0.7527
Hemoglobin (g/dL)	12.42 ± 2.14	12.14 ± 2.11	0.7654
Hematocrit (L/L)	0.38 ± 0.06	0.38 ± 0.06	0.7778
Mean corpuscular volume (fL)	84.84 ± 8.22	84.31 ± 8.54	0.9471
Mean corpuscular hemoglobin (pg)	27.30 ± 3.41	27.32 ± 3.81	0.9853
Mean corpuscular hemoglobin concentration (g/L)	322.35 ± 15.29	324.20 ± 24.53	0.7527
Red blood cell distribution width (%)	14.63 ± 3.74	15.53 ± 3.47	0.4409
Platelet count (10^9^/L)	308.38 ± 124.66	339.85 ± 118.86	0.7527
Aspartate transaminase (U/L)	25.86 ± 21.03	26.37 ± 16.12	0.9853
Alanine transaminase (U/L)	21.75 ± 12.52	23.10 ± 13.35	0.7654
Gamma-glutamyl transferase (U/L)	68.33 ± 117.56	97.24 ± 167.79	0.7527
Plasma glucose (mmol/L)	5.45 ± 0.86	6.62 ± 1.71	0.0018
Creatinine (µmol/L)	76.99 ± 19.19	82.33 ± 22.41	0.7527
Estimated glomerular filtration rate \left(\frac{\text{mL}}{\min \hspace{.25em}\cdot 1.73\hspace{.25em}{\text{m}}^{2}}\right)]	82.17 ± 16.81	78.23 ± 18.30	0.7527
AJCC staging [25]			
Stage I	19 (23.2%)	9 (34.6%)	0.4021
Stage II	23 (28.0%)	4 (15.4%)	
Stage III	20 (24.4%)	4 (15.4%)	
Stage IV	20 (24.4%)	9 (34.6%)	
Side of CRC			
Left-sided	60 (73.2%)	15 (57.7%)	0.2987
Right-sided	22 (26.8%)	11 (42.3%)	
Chemotherapy			
None	37 (45.1%)	16 (61.5%)	0.0560^**^
Adjuvant	21 (25.6%)	2 (7.7%)	
First-line	12 (14.6%)	0 (0.0%)	
Second-line	7 (8.5%)	7 (26.9%)	
Third or later-line	5 (6.1%)	1 (3.8%)	
Radiotherapy	14 (17.1%)	3 (11.5%)	0.7568
Usage of biological therapy	15 (18.3%)	7 (26.9%)	0.4611
Known comorbidities			
Hypertension	48 (58.5%)	20 (76.9%)	0.2851
Major cardiovascular event(s) before CRC	16 (19.5%)	6 (23.1%)	0.7809
Platelet aggregation inhibition	14 (17.1%)	9 (34.6%)	0.2851^*^

T2DM: type 2 diabetes mellitus. ^*^Without *p*-value correction, the differences are marginal (white blood cells: *p* = 0.0843; neutrophils: *p* = 0.0919; eosinophils: *p* = 0.0648; monocytes: *p* = 0.0918; platelet aggregation inhibition therapy: *p* = 0.0959). ^**^Without *p*-value correction, the differences in the distribution of chemotherapy regimens is statistically significant (*p* = 0.0070).

Voluntary non-CRC subjects were divided into three cohorts: 51, 50, and 65 subjects were assigned to the “*Young controls*,” “*Control*,” and “*T2DM*” groups, respectively. Duration of diabetes was 14.91 ± 9.50 within the *T2DM* group. Anamnestic, clinical, and laboratory parameters of control groups are shown in Table 2. Comparison between the volunteer groups revealed that, as expected, *Young controls* had the lowest body mass index (*p* < 0.0001 vs *T2DM* and *Controls*), systolic blood pressure (*p* = 0.0250 vs *Controls*, *p* = 0.0410 vs *T2DM*), mean corpuscular volume (*p* = 0.0002 vs *Controls*, *p* = 0.0041 vs *T2DM*), red blood cell distribution width (*p* = 0.0023 vs *Control*s, *p* < 0.0001 vs *T2DM*), fasting plasma glucose (*p* < 0.0001 vs *Controls* and *T2DM*), and the highest heart rate (*p* = 0.0002 vs *Controls*, *p* = 0.0242 vs *T2DM*) and estimated glomerular filtration rate (*p* < 0.0001 vs *Controls* and *T2DM*). T2DM patients had the highest body mass index (*p* = 0.0098 vs *Controls*, *p* < 0.0001 vs *Young controls*), white blood cell count (*p* = 0.0430 vs *Controls*, *p* = 0.0008 vs *Young controls*), gamma-glutamyl transferase (*p* = 0.0633 vs *Controls*, *p* = 0.0003 vs *Young controls*) and fasting plasma glucose (*p* < 0.0001 vs *Controls* and *Young controls*). Significant differences of comparisons among all five groups have been indicated in Table 2.

**Table 2:** Metabolic, clinical, and other parameters of voluntary control subjects

Parameter	Young controls	Control	T2DM
	(*n* = 51)	(*n* = 50)	(*n* = 65)
Age (years)	26.13 ± 4.50^1^	60.31 ± 11.00^4^	64.68 ± 8.30^7^
Sex (male:female)	24: 27	22: 28^7^	35: 30
	(47.1:52.9%)	(44.0:56.0%)	(53.8:46.2%)
Body mass index (kg/m^2^)	23.25 ± 3.95^1^	27.99 ± 5.42^6^	30.73 ± 5.12^1^
Systolic blood pressure (mmHg)	129.82 ± 15.60	138.27 ± 17.34	136.58 ± 16.64
Diastolic blood pressure (mmHg)	77.27 ± 9.10	80.98 ± 11.85	76.06 ± 10.83
Heart rate (1/min)	77.59 ± 11.08	67.76 ± 7.95^1^	73.23 ± `9.18
White blood cell count (10^9^/L)	6.48 ± 1.59^2^	7.04 ± 2.00^7^	7.97 ± 2.23
Neutrophil count (10^9^/L)	3.66 ± 1.37^2^	4.35 ± 1.68^4^	4.93 ± 1.82^7^
Eosinophil count (10^9^/L)	0.19 ± 0.17	0.18 ± 0.13	0.21 ± 0.13
Basophil count (10^9^/L)	0.06 ± 0.08	0.06 ± 0.03	0.06 ± 0.05
Monocyte count (10^9^/L)	0.44 ± 0.15^2^	0.44 ± 0.11^2^	0.52 ± 0.14
Lymphocyte count (10^9^/L)	2.09 ± 0.66^3^	2.15 ± 0.77^3^	2.25 ± 0.80^3^
Red blood cell count (10^12^/L)	4.97 ± 0.53^4^	4.92 ± 0.53^4^	4.81 ± 0.38^4^
Hemoglobin (g/dL)	14.60 ± 1.35^4^	14.69 ± 1.38^4^	14.07 ± 1.04^4^
Hematocrit (L/L)	0.43 ± 0.04^4^	0.44 ± 0.04^4^	0.42 ± 0.03^4^
Mean corpuscular volume (fL)	86.27 ± 3.00^5^	89.32 ± 4.18^3^	88.04 ± 3.44
Mean corpuscular hemoglobin (pg)	29.49 ± 1.39^3^	30.02 ± 1.61^4^	29.32 ± 1.47^3^
Mean corpuscular hemoglobin concentration (g/L)	339.16 ± 9.73^2^	337.18 ± 8.40^4^	332.95 ± 8.93^3^
Red blood cell distribution width (%)	12.59 ± 0.79^1^	13.15 ± 0.92^7^	13.23 ± 0.83^7^
Platelet count (10^9^/L)	267.86 ± 50.18	272.18 ± 72.66	263.92 ± 67.23
Aspartate transaminase (U/L)	27.47 ± 13.96	24.64 ± 6.49	25.34 ± 9.82
Alanine transaminase (U/L)	25.08 ± 14.77	24.88 ± 11.32^3^	27.91 ± 14.86
Gamma-glutamyl transferase (U/L)	25.06 ± 12.56^6^	32.70 ± 31.24	40.64 ± 29.40
Plasma glucose (mmol/L)	4.58 ± 0.45^1^	4.99 ± 0.57^1^	8.17 ± 2.63^1^
Creatinine (µmol/L)	73.69 ± 12.72	70.20 ± 12.72	75.11 ± 19.05
Estimated glomerular filtration rate \left(\frac{\text{mL}}{\min \hspace{.25em}\cdot 1.73\hspace{.25em}{\text{m}}^{2}}\right)]	109.33 ± 14.97^1^	89.66 ± 12.42	83.98 ± 16.05
Known comorbidities			
Hypertension	0 (0.0%)^1^	16 (32.0%)^1^	56 (86.2%)^3^
Major cardiovascular event(s) prior visit date	0 (0.0%)^2^	5 (10.0%)	13 (20.0%)
Platelet aggregation inhibition	0 (0.0%)^2^	6 (12.0%)^6^	50 (76.9%)^4^

T2DM: type 2 diabetes mellitus, ^1^ *p* < 0.05 vs all other four groups, ^2^ *p* < 0.05 vs all diseased groups, ^3^ *p* < 0.01 vs the CRC without T2DM group, ^4^ *p* < 0.01 vs both tumor groups, ^5^ *p* < 0.05 vs Control and T2DM groups, ^6^ *p* < 0.05 vs T2DM, and ^7^ *p* < 0.01 vs the CRC with T2DM group.

The duration of diabetes was shorter within the *CRC + T2DM* group compared to those of the *T2DM* group. A higher proportion of oral antidiabetic drug usage and diet-only therapy was observable within the *CRC + T2DM* group, whereas the need for insulin therapy was greater in those within the *T2DM* group (*p* = 0.0006). Furthermore, lower glycated hemoglobin (HbA_1C_) level and fewer diabetic complications were found in the *CRC + T2DM* patients (Table 3). The occurrence of hypertension (*p* = 0.7790) and the proportion of previous major cardiovascular event(s) before the first visit (*p* = 0.7004) did not differ between the two diabetic groups. Comparison of diabetes-related parameters is summarized in Table 3.

**Table 3:** Diabetes-related parameters of T2DM and *CRC + T2DM* patients

Parameter	T2DM	*CRC + T2DM*	*p*-value
	(*n* = 65)	(*n* = 26)	
Duration of T2DM (years)	14.91 ± 9.50	6.88 ± 6.10	0.0015
HbA_1C_ (%)	7.40 ± 1.26	6.30 ± 1.04	0.0012
Treatment used for T2DM			
Only diet	3 (4.6%)	7 (26.9%)	0.0006
Oral hypoglycemic medications	32 (49.2%)	18 (69.2%)	
Combination therapy (oral + basal insulin)	14 (21.5%)	1 (3.8%)	
Intensive insulin therapy	16 (24.6%)	0 (0.0%)	
Diabetic complications^1^			
Retinopathy	16 (24.6%)	2 (7.7%)	0.4203
Nephropathy	6 (9.2%)	0 (0.0%)	0.5319
Neuropathy	14 (21.5%)	5 (19.2%)	1.0000
Angiopathy	9 (13.8%)	1 (3.8%)	0.5420
Albuminuria	9 (13.8%)	0 (0.0%)	0.3330^2^
Number of diabetic comorbidities			
None	35 (53.8%)	20 (76.9%)	0.4203
One	15 (23.1%)	4 (15.4%)	
More than one	15 (23.1%)	2 (7.7%)	
Hyperlipidemia	44 (67.7%)	10 (38.5%)	0.1210^3^

HbA_1C_: glycated hemoglobin, ^1^All developed prior CRC, ^2^Without *p*-value correction *p* = 0.0555, and ^3^Without *p*-value correction *p* = 0.0173.

### 3.1 Baseline measurement of paraneoplastic thrombocytosis-related parameters

**Figure 1:**
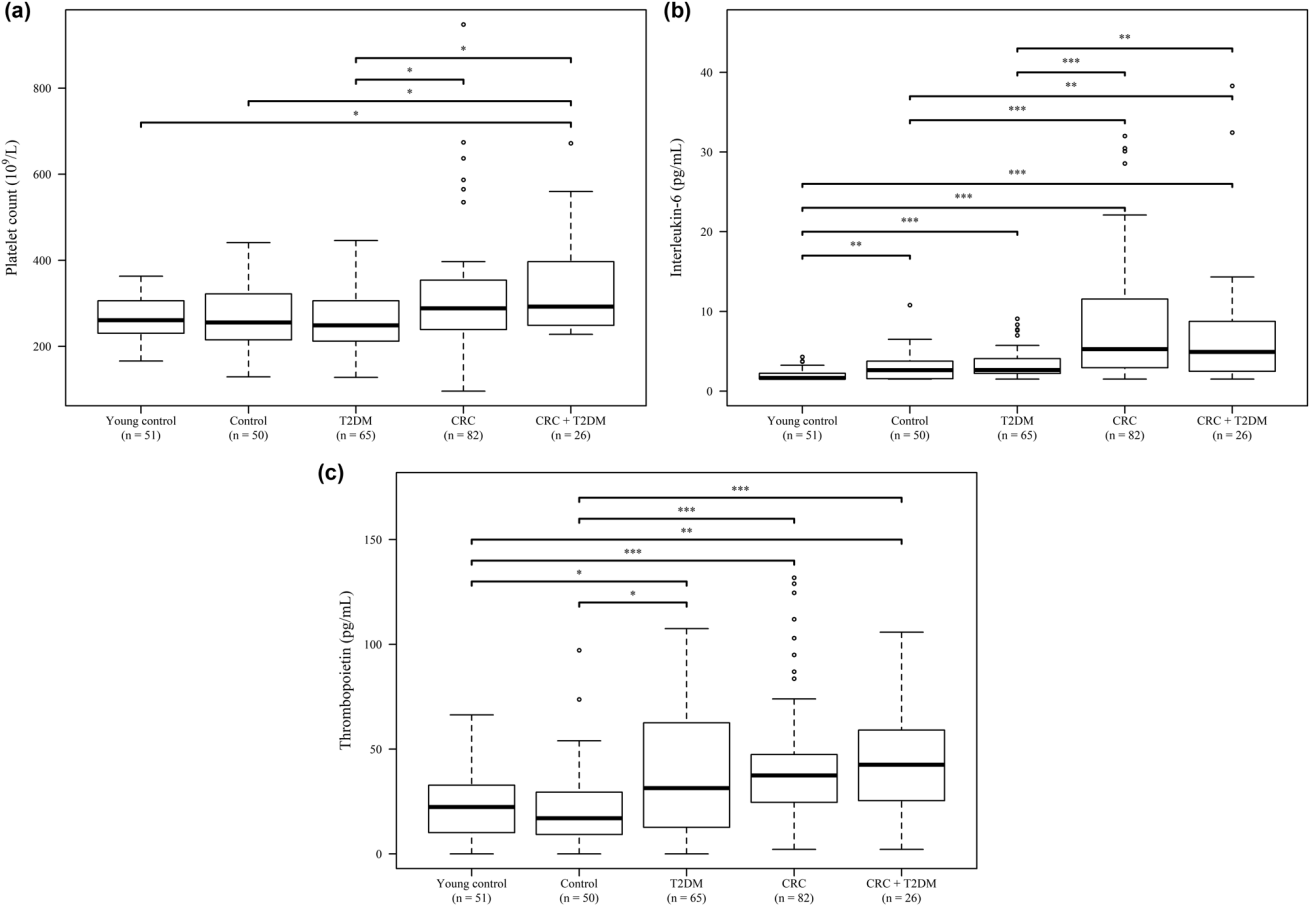
Comparison of paraneoplastic thrombocytosis-related parameters within the five study groups. (a) The platelet count was significantly higher in CRC patients with T2DM, compared to those in all control groups, and it also differed between the *CRC* and *T2DM* groups. (b) The plasma interleukin-6 level was significantly higher in all tumor patients^1^, whereas (c) the plasma thrombopoietin level was significantly lower within the *T2DM* and *CRC*, and significantly higher in the *CRC + T2DM* groups, compared to those of control subjects. Note: *p*-value correction was used for all between-groups comparisons. ^1^Due to better visibility, six and one outlier(s) over 40 pg/mL were not shown in the *CRC* and *CRC + T2DM* groups; maximum values were 220.20 and 77.19 pg/mL, respectively.

Plasma level of paraneoplastic thrombocytosis parameters of patients was compared to those of all control groups at the time of CRC diagnosis. Highest platelet counts were observed within the two tumor groups: platelet count of *CRC* patients was significantly higher than those of within the *T2DM* group (*p* = 0.0369), whereas the platelet count of the *CRC + T2DM* group was significantly higher than all of the control groups (*p* = 0.0369 vs *Young Control* and *Control*, *p* = 0.0278 vs *T2DM*; Figure 1a). Lowest plasma interleukin-6 levels were observed within *Young controls* (*p* < 0.0010 vs all other cohorts). Subjects of the *Control* and *T2DM* groups had similar interleukin-6 levels; and interleukin-6 was significantly higher in both tumor groups, compared to all of those observed in control groups (*p* < 0.0001 *CRC* vs *Control* and *T2DM*, *p* = 0.0011 *CRC + T2DM* vs *Control*, and *p* = 0.0069 *CRC + T2DM* vs *T2DM*; Figure 1b). Thrombopoietin level was basically the same in the *Young control* and *Control* groups, and a separate cluster was formed by the remaining three groups. The highest thrombopoietin levels were observed in the *CRC + T2DM* group (Figure 1c). No further difference could have been justified in any of the study groups if they were further subdivided by the usage of platelet aggregation inhibition therapy, antidiabetic drugs, or in the presence of any diabetic complications.

Highest platelet counts were found if interleukin-6 was high as well (*CRC*: Spearman *ρ*: +0.34, *p* = 0.0017; *CRC + T2DM*: *p* = 0.0786; CRC groups combined: Spearman *ρ*: +0.32, *p* = 0.0009). Correlation between platelet count and thrombopoietin levels was only significant in the *CRC* group (Spearman *ρ*: –0.24, *p* = 0.0332), whereas no (*p* = 0.8486) and marginal association (*p* = 0.0643) was found in the *CRC + T2DM* and in the two tumor groups combined, respectively. No correlation was found between interleukin-6 and thrombopoietin levels (*CRC*: *p* = 0.4279, *CRC + T2DM*: *p* = 0.9921, tumor groups combined: *p* = 0.5383). No correlation was found between the thrombocytosis-related parameters and the duration of T2DM or the preoperative level of HbA_1C_.

### 3.2 Changes in the parameters of paraneoplastic thrombocytosis with the course of CRC

**Figure 2:**
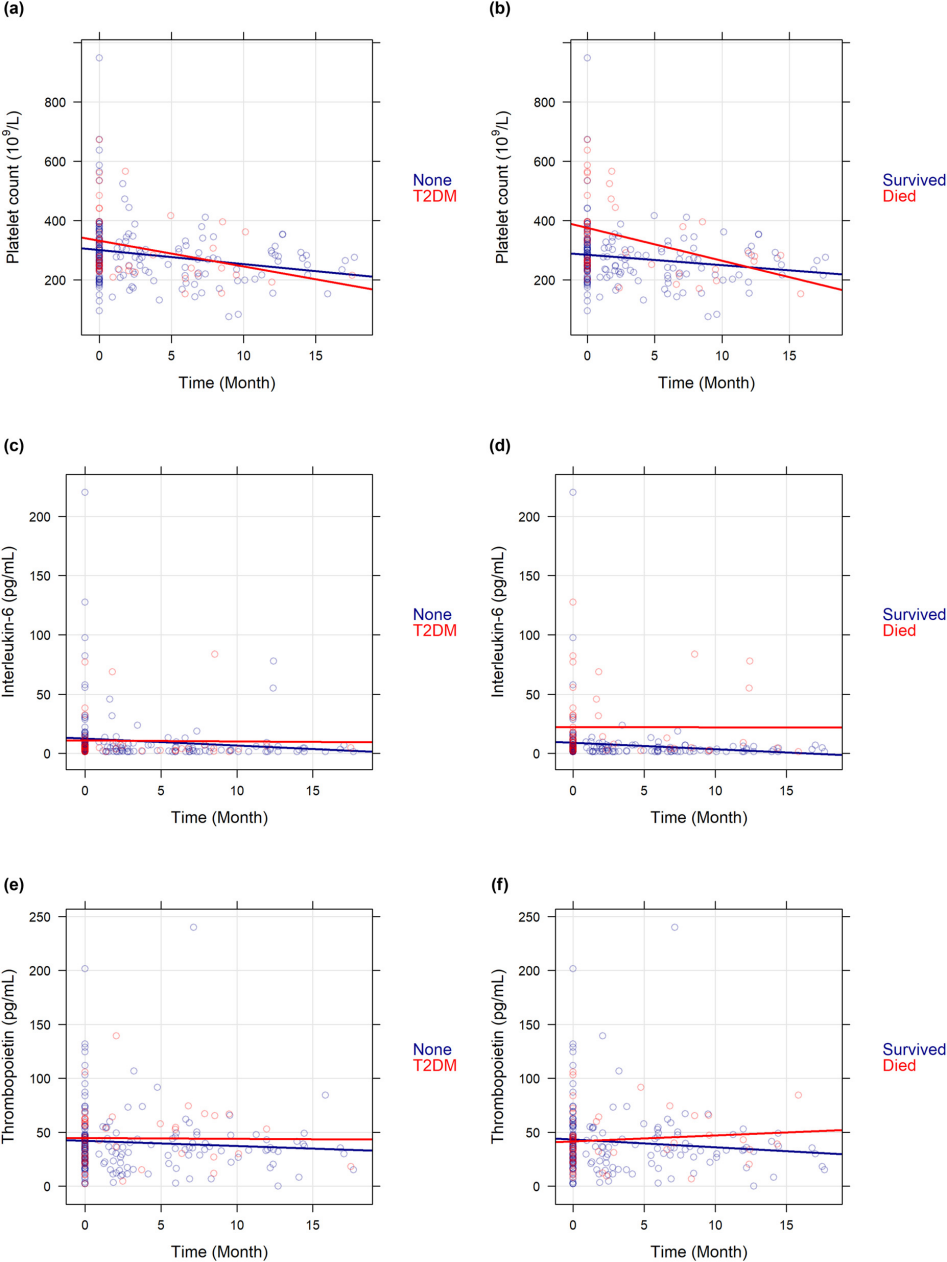
Change in the parameters of paraneoplastic thrombocytosis with the course of the disease. No difference was found in any of the parameters (a, c, e) when stratified with the presence of T2DM. (b) Platelet count and (d) plasma interleukin-6 were significantly higher at the time of CRC diagnosis in those patients who died during the observation period. Within the first year after tumor removal surgery, a significantly faster platelet count decrease was found in those patients who died (b), whereas the significant difference in plasma interleukin-6 levels remained throughout the whole study between survivors and those patients who died (d). No statistical difference was found in plasma thrombopoietin levels of survivors and nonsurvivors (*p* = 0.5940, f).

For the 108 CRC subjects, a total of 215 measurements were available. 108, 48, 37, and 22 preoperative, postoperative, 6-month, and 12-month measurements were available, respectively. Significant decrease in later measurements occurred due to the death of patients, disease progression resulting in higher ECOG performance status and patient’s unavailability to attend at later visits, the need to initiate chemotherapy earlier than the postoperative visit window, or continuous chemotherapy without drug holiday after the second study visit. Due to the decreasing number of follow-ups, a more robust statistical method not sensitive to the loss of follow-up had to be chosen; therefore, it was investigated via age-corrected and stage-corrected linear mixed effect interaction models as to how T2DM and worse clinical outcome (death) affect the changes of platelet count, interleukin-6, and thrombopoietin levels with the course of the disease. Average survival time: 16.96 ± 11.43 months, all within the first 3 years after CRC diagnosis. Diabetes had no significant effect on any of the changes (platelet count: *p* = 0.1190; interleukin-6: *p* = 0.5571; thrombopoietin: *p* = 0.3062, Figure 2a, c, and e). Platelet counts of all patients decreased, but among those patients who died during the time of the study, the average baseline platelet count was significantly higher (273.70 vs 353.00 × 10^9^/L; *p* = 0.0042) and a faster decrease could have been seen within the first 12 months after the tumor removal surgery compared to those of who survived (Figure 2b). Similarly, increased baseline interleukin-6 levels (5.76 vs 27.42 pg/mL; *p* < 0.0001) and marginally faster decreasing levels (*p* = 0.0613) were observed in deceased patients over time, whereas in those who survived the plasma interleukin-6 level was constant during our observation (*p* = 0.1273, Figure 2d). The initial thrombopoietin level did not differ between surviving and deceased patients (*p* = 0.5747), and its change was not affected by the worse clinical outcome (*p* = 0.5940, Figure 2f). Usage of radiotherapy and chemotherapy did not affect any of the response variables, if included within any of the models.

### 3.3 Survival analysis of paraneoplastic thrombocytosis-related parameters and T2DM

**Figure 3:**
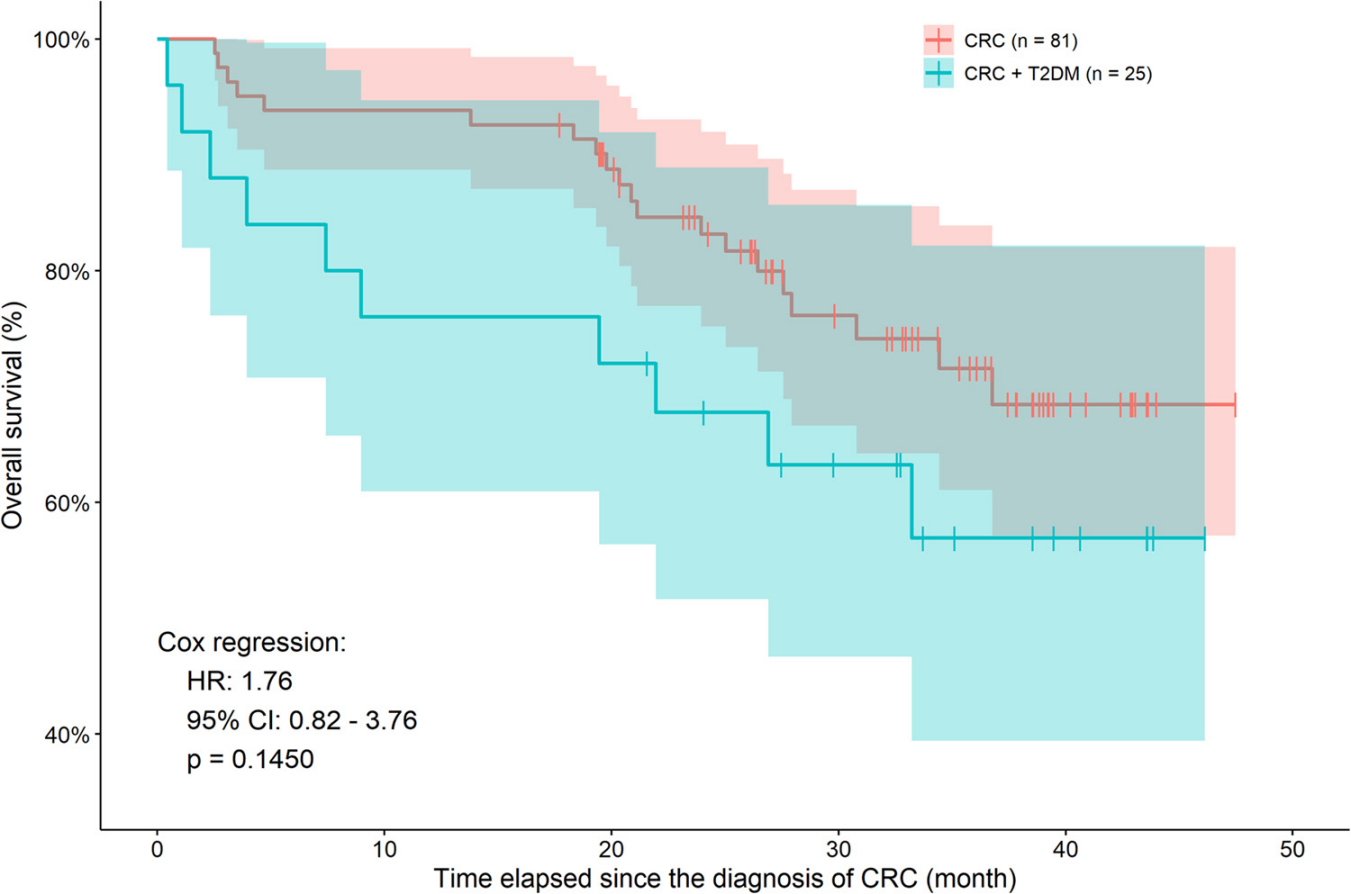
Naïve Kaplan–Meier curves of CRC patients with and without T2DM. It should be noted that although the two curves do not appear to be similar, neither Cox regression (*p* = 0.1450), nor log-rank test (*p* = 0.0908) could justify a statistical difference.

Thirty of the 108 patients (27.8%) died during the study, from which 20 and 10 belonged to the *CRC* and *CRC + T2DM* group, respectively. Patients were followed up no later than January 31, 2021. The survival analysis was performed on both the preoperative (single-time) and longitudinal data. Despite the higher occurrence of death (24 vs 40%) within the diabetic tumor group and the difference that appears to be significant on the naïve Kaplan–Meier figure (Figure 3), the univariate Cox model of preoperative data suggested that T2DM had no effect on patient survival (*p* = 0.1450). A higher preoperative platelet count (HR: 1.0026, 95% CI: 1.0010–1.0050, *p* = 0.0052) could be considered as a poor prognostic sign. Interleukin-6 and thrombopoietin levels had no significant univariate effect; however, if combined with T2DM in a multivariate model, higher interleukin-6 levels had marginal effect (HR: 1.0007, 95% CI: 0.9995–1.0140, *p* = 0.0692) on patient survival. Similar to the univariate model, higher platelet counts had the same significant effect in the multivariate model as well (HR: 1.0028, 95% CI: 1.0009–1.0050, *p* = 0.0043). T2DM did not have any effect in either multivariate model. A subgroup analysis within the *CRC + T2DM* group only revealed that neither the duration of diabetes (*p* = 0.5590), the use of any antidiabetics (*p* = 0.2620), the presence of any diabetic complications (*p* = 0.2860), nor the preoperative level of HbA_1C_ (*p* = 0.5370) affected patient survival.

To analyze the effect of paraneoplastic thrombocytosis-related parameter changes in time, Bayesian joint models were used. First, three univariate joint models (a single parameter is analyzed within the longitudinal submodel) were constructed: the survival effect of the changes of platelet count, interleukin-6, or thrombopoietin over time was included in the longitudinal submodel, and T2DM was included in the survival submodel. An additional multivariate joint model was also constructed, where all three paraneoplastic thrombocytosis parameters were included within the longitudinal submodel, and no change was applied to the survival submodel. Interpretation of the clinical significance of parameters on patient survival was assessed as described in Section 2.

**Figure 4:**
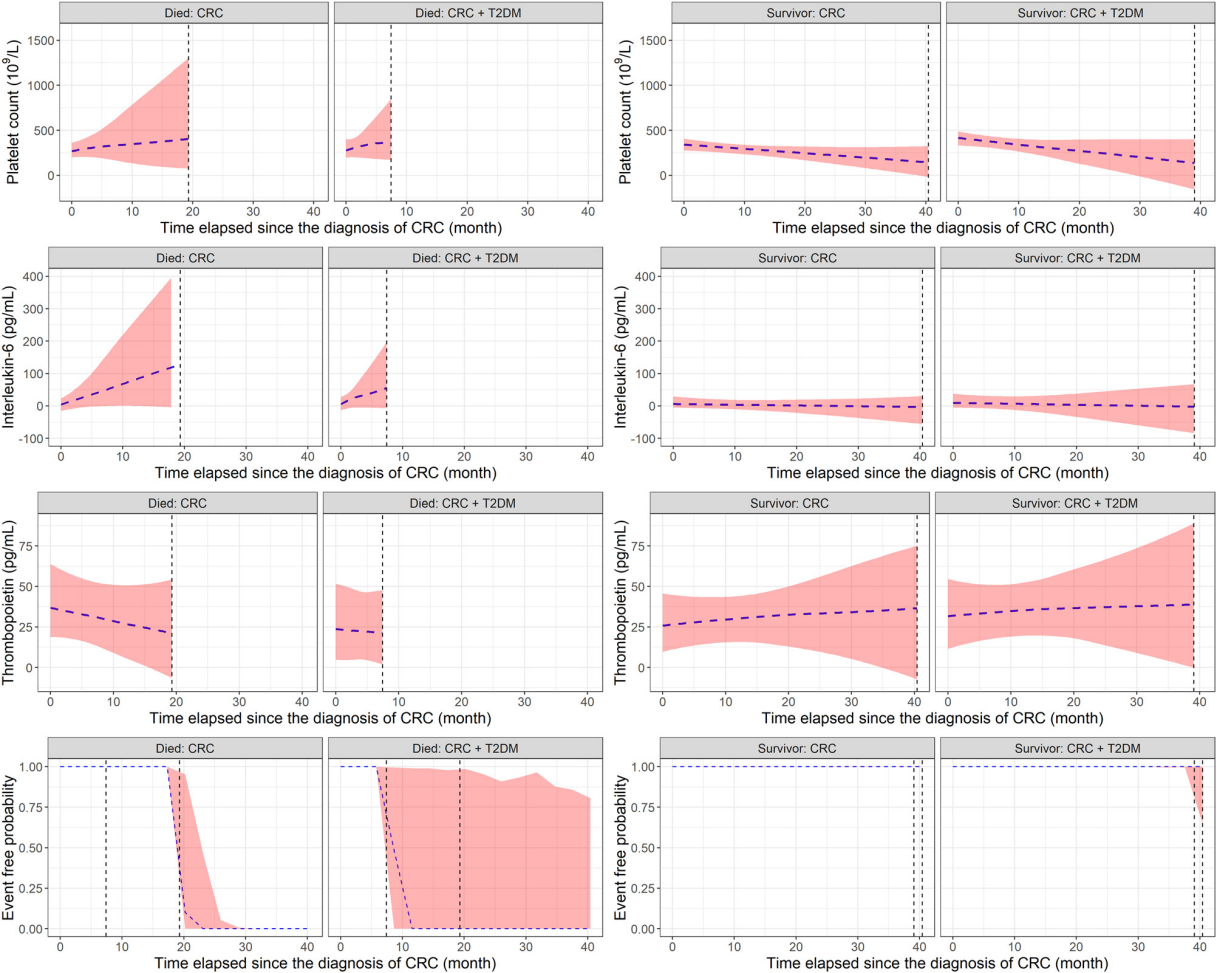
Longitudinal and survival predictions of the Bayesian joint models. No difference was found between CRC patients with or without T2DM. Increasing platelet count and plasma interleukin-6 level were associated with a higher risk of shorter survival times (left), whereas no change or a slow decline in these parameters is a good prognostic sign (right). Thrombopoietin levels had no clinically relevant effect neither in univariate nor in multivariate survival models.

**Figure 5:**
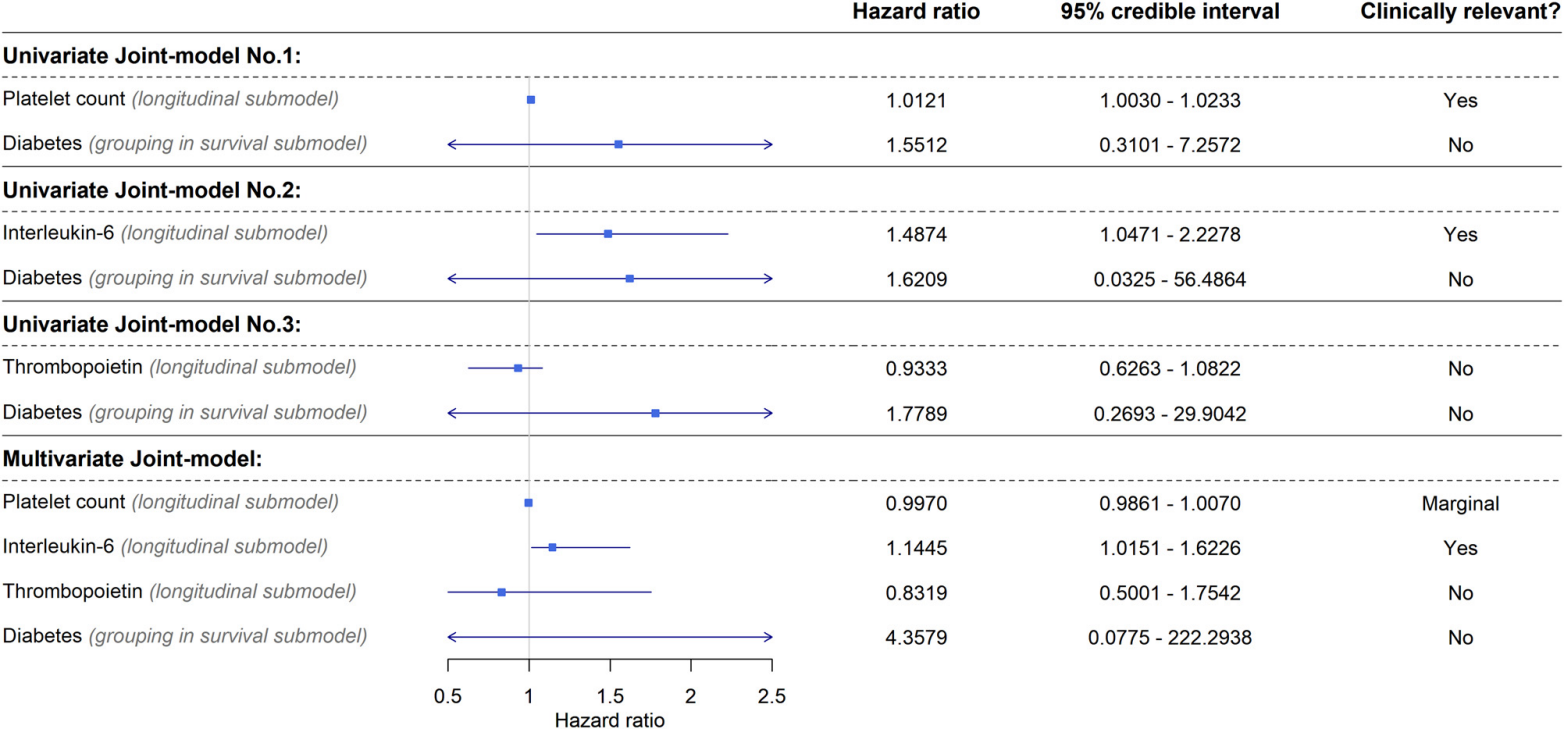
Results of the Bayesian joint survival models. Platelet count and/or plasma interleukin-6 level increasing over time is a poor prognostic factor, whereas diabetes and plasma thrombopoietin changes did not affect patient survival (Note: credible interval is the Bayesian equivalent of the frequentists’ CI.).

Based on the result of the univariate joint models, after the surgical removal of the primary tumor, higher platelet count and plasma interleukin-6 level is a sign of poorer survival (Figure 4). Thrombopoietin and diabetes did not affect the survival of patients in any of the univariate models. Multivariate joint model results suggested that interleukin-6 had the strongest effect on patient survival, whereas the platelet count was marginal; thrombopoietin and diabetes had no clinically relevant effect, similar to those observed in univariate models (Figures 4 and 5).

## 4 Discussion

In CRC, the occurrence of thrombocytosis, either prior or after the primary tumor removal surgery, is associated with shorter survival of patients [1,2,30,31,32,33]. The background of thrombocytosis may vary due to several factors, including the bleeding (reactive thrombocytosis) or metabolic changes (paraneoplastic thrombocytosis) of the tumor. In the latter, the tumor produces cytokines, such as interleukin-6, in higher quantities, and those higher cytokine concentrations stimulate hepatic thrombopoietin production, which ultimately results in the overproduction of platelets [4,34]. A platelet count of >400 × 10^9^/L or >450 × 10^9^/L is the most common definition of thrombocytosis [1,3,35], but several earlier studies reported other cut-off values [1], indicating that the definition of thrombocytosis may not be perfect. In an earlier study [20], we have reported that a personalized, relative platelet measure can predict disease outcome significantly better than “traditional” thrombocytosis, which further strengthened that the definition of thrombocytosis needs to be revised. The biochemical detection of paraneoplastic thrombocytosis in CRC is a novel research area [36]. In the current study, we observed higher interleukin-6 and thrombopoietin levels of CRC patients, compared to those of control subjects, supporting the presence of paraneoplastic thrombocytosis, which was further strengthened by the result of correlation and survival analysis, in line with earlier findings [1,2,31,32,33,36,37,38].

There is a strong relationship between CRC and various cytokines, including interleukin-6, which can play significant roles both in the development and progression of the disease [39,40,41]. Specifically, interleukin-6 is known to for its significant role in tumor proliferation, migration, induction of microsatellite instability, and angiogenesis [42,43]. It has been reported that interleukin-6 is produced by cancer-associated mesenchymal stem cells [44]. When combined with glycoprotein 130 (gp130, synonyms: IL6ST or CD130) interleukin-6 can regulate disease progression through the Shp2-Ras-ERK, JAK1/2-STAT3, and PI3K-Akt-mTOR pathways, and its higher serum levels were associated with larger tumor size, presence of metastases, and worse overall- and disease-free survival [40,41,45,46]. Due to its strong connection with CRC, interleukin-6 has been proposed as a good prognostic marker of CRC [47,48,49]. In the current study, we could also confirm the prognostic role of interleukin-6 over patient survival. The result that preoperative interleukin-6 did not affected survival was possibly due to the heterogeneity of study population, which was somewhat confirmed by the results obtained from the multivariate Cox models, as the predictive effect of interleukin-6 on survival increased. As a novel result, we found that constantly higher interleukin-6 level could have been observed in those patients, who died within the first 3 years after CRC diagnosis.

Research on CRC and thrombopoietin is limited. Earlier studies have identified that cancer cells can induce circulating thrombopoietin production [50]; furthermore, higher thrombopoietin level is associated with gastrointestinal cancers, and a possible relationship between more advanced clinical stages and thrombopoietin has been also suggested [51,52]. Similar to the observations above, we found that the thrombopoietin level of CRC patients is higher than those of healthy subjects; however, the effect of thrombopoietin levels over survival was negligible, and no change with the course of the disease could have been identified in either of our longitudinal models.

T2DM, similarly to (paraneoplastic) thrombocytosis, has a known negative effect on CRC [15,53]. Several potential mechanisms link the two diseases together [19], including the increased plasma glucose levels (hyperglycemia), the presence of insulin resistance and hyperinsulinemia, increased insulin-like growth factor-1 levels, increased oxidative stress, higher cytokine concentrations, and increased platelet activation [9,10,13,19,53]. CRC is known to have an increased incidence in T2DM patients compared to those of within the healthy population [9,10]; a 1.3-fold increased risk of CRC has been reported [54,55]. T2DM has been described to have a negative effect on overall-, cancer-specific-, disease-free-, and recurrence-free survival of CRC patients [56,57]. In contrast to earlier findings, we found that T2DM did not affect patient survival in the current study statistically, but it has to be noted, that the percentage of patients who died was higher in the *CRC + T2DM* group (24 vs 40%), and similarly, the survival curves on Figure 3 also suggested a tendency toward significant difference. The effect of T2DM on patient survival was further investigated through the presence of diabetic complications, the preoperative HbA_1C_ level, and the duration of T2DM. Despite the fact that the duration of diabetes was basically twice as long in patients of the *T2DM* group, a greater number of diabetic complications were present, and a more significant proportion of patients required (intensive) insulin therapy, no significant effect could be justified for any of the above-mentioned parameters. It has to be mentioned though that the sample size of *CRC + T2DM* patients was low, but we could not even prove tendentious differences similar to that of observed in Figure 3.

The relationship between CRC-related paraneoplastic thrombocytosis and T2DM through biochemical measurements has not been investigated previously. It has to be mentioned though, that various platelet abnormalities and increased interleukin-6 and thrombopoietin production are known in T2DM [16,17,18,58,59,60]. Due to the above-mentioned effects and the high risk of cardiovascular events, the usage of platelet aggregation inhibition therapy is very common in T2DM [61,62]. Based on the available literature, our prestudy hypothesis was that CRC patients who also suffer from T2DM would probably have higher interleukin-6 and thrombopoietin levels than those who are not affected by T2DM. In contrast to our hypothesis, no differences could have been justified in any of the parameters related to paraneoplastic thrombocytosis between the *CRC* and *CRC + T2DM* groups. Furthermore, platelet aggregation inhibition also did not have any effect on the parameters of paraneoplastic thrombocytosis even though that the therapy was more common within the *CRC + T2DM* group. Similarly, the diabetes-related parameters also did not affect the paraneoplastic thrombocytosis-related parameters. The fact that plasma thrombopoietin level of non-CRC T2DM patients was more similar to those of CRC patients than those of control subjects was most likely related to the already known fact that thrombopoietin levels are higher in T2DM [58], but no previous data are available on how similar these values of CRC and T2DM patients should be. The presented data suggest that T2DM does not increase the effect of CRC-related paraneoplastic thrombocytosis, in most probability, and the disease-worsening effect of T2DM, which has been described in earlier publications [11], must be carried out through other factors. To identify those factors, further investigations are needed.

### 4.1 Limitations

Limitations of the current study were the small sample size and the heterogeneity of CRC population – the latter may be also compensated with a larger sample size. The proportion of T2DM patients within the tumor cohort corresponded to the healthy Hungarian population (approximately every fourth person, over 60 years of age [8]). Due to patients’ decision, a large number of potential subjects did not agree to be included in the study. Despite the low number of cases, it is important to emphasize that the presence of paraneoplastic thrombocytosis could have been already detected at such a low number of cases, showing its significance in CRC. The low number of T2DM in those of CRC patients allowed us only to pinpoint tendentious differences in several parameters.

## 5 Conclusion

To summarize the results of the current study, our data suggested that although some metabolic changes do occur to platelet counts and to the interleukin-6 and/or thrombopoietin synthesis in T2DM and in paraneoplastic thrombocytosis-affected CRC patients, no combined effect have been observed. Based on the current study, there is no significant relationship between the two conditions with high probability and the known disease-worsening effect of diabetes on CRC survival is presumably independent of paraneoplastic thrombocytosis.

## References

[1] Baranyai Z, Josa V, Toth A, Szilasi Z, Tihanyi B, Zarand A, et al. Paraneoplastic thrombocytosis in gastrointestinal cancer. Platelets. 2016;27(4):269–75. 10.3109/09537104.2016.1170112. 27136385

[2] Josa V, Krzystanek M, Eklund AC, Salamon F, Zarand A, Szallasi Z, et al. Relationship of postoperative thrombocytosis and survival of patients with colorectal cancer. Int J Surg. 2015;18:1–6. 10.1016/j.ijsu.2015.03.005. 25843227

[3] Stone RL, Nick AM, McNeish IA, Balkwill F, Han HD, Bottsford-Miller J, et al. Paraneoplastic thrombocytosis in ovarian cancer. N Engl J Med. 2012;366(7):610–8. 10.1056/NEJMoa1110352. PMC3296780 22335738

[4] Lin RJ, Afshar-Kharghan V, Schafer AI. Paraneoplastic thrombocytosis: the secrets of tumor self-promotion. Blood. 2014;124(2):184–7. 10.1182/blood-2014-03-562538. PMC4093679 24868077

[5] International Diabetes Federation: IDF Diabetes Atlas, 9th edition. Brussels, Belgium: International Diabetes Federation; 2019 [cited 2020 Apr 15]. Available from: https://www.diabetesatlas.org

[6] DeFronzo RA, Ferrannini E, Groop L, Henry RR, Herman WH, Holst JJ, et al. Type 2 diabetes mellitus. Nat Rev Dis Prim. 2015;1:15019. 10.1038/nrdp.2015.19. 27189025

[7] Sung H, Ferlay J, Siegel LR, Laversanne M, Soerjomataram I, Jemal A, et al. Global cancer statistics 2020: GLOBOCAN estimates of incidence and mortality worldwide for 36 cancers in 185 countries. CA Cancer J Clin. 2021;71(3):209–49. 10.3322/caac.21660. 33538338

[8] Kempler P, Putz Z, Kiss Z, Wittmann I, Abonyi-Tóth Z, Gy R, et al. Prevalence and financial burden of type 2 diabetes mellitus in Hungary between 2001–2014 – results of the analysis of the national health insurance fund database. Diab Hung. 2016;24(3):177–88. Hungarian.

[9] Tsilidis KK, Kasimis JC, Lopez DS, Ntzani EE, Ioannidis JP. Type 2 diabetes and cancer: umbrella review of meta-analyses of observational studies. BMJ. 2015;350:g7607. 10.1136/bmj.g7607. 25555821

[10] Giovannucci E, Harlan DM, Archer MC, Bergenstal RM, Gapstur SM, Habel LA, et al. Diabetes and cancer: a consensus report. CA Cancer J Clin. 2010;60(4):207–21. 10.3322/caac.20078. 20554718

[11] Ling S, Brown K, Miksza JK, Howells L, Morrison A, Issa E, et al. Association of type 2 diabetes with cancer: a meta-analysis with bias analysis for unmeasured confounding in 151 cohorts comprising 32 million people. Diabetes Care. 2020;43(9):2313–22. 10.2337/dc20-0204. 32910779

[12] Siegel RL, Miller KD, Jemal A. Cancer statistics, 2016. CA Cancer J Clin. 2016;66(1):7–30. 10.3322/caac.21332. 26742998

[13] Gonzalez N, Prieto I, Del Puerto-Nevado L, Portal-Nunez S, Ardura JA, Corton M, et al. 2017 update on the relationship between diabetes and colorectal cancer: epidemiology, potential molecular mechanisms and therapeutic implications. Oncotarget. 2017;8(11):18456–85. 10.18632/oncotarget.14472. PMC5392343 28060743

[14] Hippisley-Cox J, Coupland C. Development and validation of risk prediction equations to estimate survival in patients with colorectal cancer: cohort study. BMJ. 2017;357:j2497. 10.1136/bmj.j2497. PMC5471851 28620089

[15] Li J, Liu J, Gao C, Liu F, Zhao H. Increased mortality for colorectal cancer patients with preexisting diabetes mellitus: an updated meta-analysis. Oncotarget. 2017;8(37):62478–88. 10.18632/oncotarget.19923. PMC5617522 28977962

[16] Ferreiro JL, Gomez-Hospital JA, Angiolillo DJ. Platelet abnormalities in diabetes mellitus. Diab Vasc Dis Res. 2010;7(4):251–9. 10.1177/1479164110383994. 20921090

[17] Yazbek N, Bapat A, Kleiman N. Platelet abnormalities in diabetes mellitus. Coron Artery Dis. 2003;14(5):365–71. 10.1097/01.mca.0000085138.16622.9e. 12878901

[18] Lee RH, Bergmeier W. Sugar makes neutrophils RAGE: linking diabetes-associated hyperglycemia to thrombocytosis and platelet reactivity. J Clin Invest. 2017;127(6):2040–3. 10.1172/JCI94494. PMC5451221 28504654

[19] Shlomai G, Neel B, LeRoith D, Gallagher EJ. Type 2 diabetes mellitus and cancer: the role of pharmacotherapy. J Clin Oncol. 2016;34(35):4261–9. 10.1200/JCO.2016.67.4044. PMC5455318 27903154

[20] Herold Z, Herold M, Lohinszky J, Dank M, Somogyi A. Personalized indicator thrombocytosis shows connection to staging and indicates shorter survival in colorectal cancer patients with or without type 2 diabetes. Cancers (Basel). 2020;12(3):556. 10.3390/cancers12030556. PMC7139544 32121060

[21] Jardim DL, Rodrigues CA, Novis YAS, Rocha VG, Hoff PM. Oxaliplatin-related thrombocytopenia. Ann Oncol. 2012;23(8):1937–42. 10.1093/annonc/mds074. 22534771

[22] Kilpatrick K, Shaw JL, Jaramillo R, Toler A, Eisen M, Sangare L, et al. Occurrence and management of thrombocytopenia in metastatic colorectal cancer patients receiving chemotherapy: secondary analysis of data from prospective clinical trials. Clin Colorectal Cancer. 2020;20(2):170–6. 10.1016/j.clcc.2020.10.004. 33281065

[23] Schwandt A, Denkinger M, Fasching P, Pfeifer M, Wagner C, Weiland J, et al. Comparison of MDRD, CKD-EPI, and Cockcroft-Gault equation in relation to measured glomerular filtration rate among a large cohort with diabetes. J Diabetes Complications. 2017;31(9):1376–83. 10.1016/j.jdiacomp.2017.06.016. 28711195

[24] Shen H, Yang J, Huang Q, Jiang MJ, Tan YN, Fu JF, et al. Different treatment strategies and molecular features between right-sided and left-sided colon cancers. World J Gastroenterol. 2015;21(21):6470–8. 10.3748/wjg.v21.i21.6470. PMC4458758 26074686

[25] Jessup J, Goldberg R, Asare E, Benson A, Brierley J Chang G, et al. Colon and rectum. In Amin M, Edge S, Greene F, Byrd D, Brookland R, Washington M, et al., editors. AJCC Cancer Staging Manual. 8th edn. Chicago, IL, USA: Springer International Publishing; 2018. p. 251–74.

[26] R Core Team. R: a language and environment for statistical computing. Vienna, Austria: R Foundation for Statistical Computing; 2021. Available from: https://www.R-project.org/

[27] Pinheiro J, Bates D, DebRoy S, Sarkar D, R Core Team. {nlme}: linear and nonlinear mixed effects models (R package version 3.1-149). 2021; Available from: https://CRAN.R-project.org/package=nlme

[28] Goodrich B, Gabry J, Ali I, Brilleman S. rstanarm: Bayesian applied regression modeling via Stan (R package version 2.21.1). 2020; Available from: https://mc-stan.org/rstanarm

[29] Holm S. A simple sequentially rejective multiple test procedure. Scand J Stat. 1979;6(2):65–70.

[30] Baranyai Z, Josa V, Krzystanek M, Eklund AC, Szasz AM, Szallasi Z. Evaluation of thrombocytosis as predictive factor in colorectal cancer. Magy Seb. 2013;66(6):331–7. 10.1556/MaSeb.66.2013.6.5. (Hungarian). 24333978

[31] Gu D, Szallasi A. Thrombocytosis portends adverse prognosis in colorectal cancer: a meta-analysis of 5,619 patients in 16 individual studies. Anticancer Res. 2017;37(9):4717–26. 10.21873/anticanres.11878. 28870890

[32] Ishizuka M, Nagata H, Takagi K, Iwasaki Y, Kubota K. Preoperative thrombocytosis is associated with survival after surgery for colorectal cancer. J Surg Oncol. 2012;106(7):887–91. 10.1002/jso.23163. 22623286

[33] Ramjeesingh R, Jones A, Orr C, Bricks CS, Hopman WM, Hammad N. Thrombocytosis as a predictor of poor prognosis in colorectal cancer patients. J Clin Oncol. 2016;34(S4):540. 10.1200/jco.2016.34.4_suppl.540.

[34] Bleeker JS, Hogan WJ. Thrombocytosis: diagnostic evaluation, thrombotic risk stratification, and risk-based management strategies. Thrombosis. 2011;2011:536062. 10.1155/2011/536062. PMC3200282 22084665

[35] Wille K, Sadjadian P, Griesshammer M. Thrombocytosis and thrombocytopenia – background and clinical relevance. Dtsch Med Wochenschr. 2017;142(23):1732–43. 10.1055/s-0042-111096. (German). 29145678

[36] Josa V, Brodszky V, Zarand A, Mezei T, Szilasi Z, Merkel K, et al. The relationship between IL-6 and thrombocytosis accompanying gastrointestinal tumours. Prz Gastroenterol. 2020;15(3):215–9. 10.5114/pg.2020.98538. PMC7509901 33005266

[37] Cravioto-Villanueva A, Luna-Perez P, Gutierrez-de la Barrera M, Martinez-Gomez H, Maffuz A, Rojas-Garcia P, et al. Thrombocytosis as a predictor of distant recurrence in patients with rectal cancer. Arch Med Res. 2012;43(4):305–11. 10.1016/j.arcmed.2012.06.008. 22727694

[38] Voutsadakis IA. Thrombocytosis as a prognostic marker in gastrointestinal cancers. World J Gastrointest Oncol. 2014;6(2):34–40. 10.4251/wjgo.v6.i2.34. PMC3926972 24567794

[39] West NR, McCuaig S, Franchini F, Powrie F. Emerging cytokine networks in colorectal cancer. Nat Rev Immunol. 2015;15(10):615–29. 10.1038/nri3896. 26358393

[40] Mager LF, Wasmer MH, Rau TT, Krebs P. Cytokine-induced modulation of colorectal cancer. Front Oncol. 2016;6:96. 10.3389/fonc.2016.00096. PMC4835502 27148488

[41] Li J, Huang L, Zhao H, Yan Y, Lu J. The role of interleukins in colorectal cancer. Int J Biol Sci. 2020;16(13):2323–39. 10.7150/ijbs.46651. PMC7378639 32760201

[42] Taniguchi K, Karin M. IL-6 and related cytokines as the critical lynchpins between inflammation and cancer. Semin Immunol. 2014;26(1):54–74. 10.1016/j.smim.2014.01.001. 24552665

[43] Tseng-Rogenski SS, Hamaya Y, Choi DY, Carethers JM. Interleukin 6 alters localization of hMSH3, leading to DNA mismatch repair defects in colorectal cancer cells. Gastroenterology. 2015;148(3):579–89. 10.1053/j.gastro.2014.11.027. PMC4339542 25461668

[44] Lin JT, Wang JY, Chen MK, Chen HC, Chang TH, Su BW, et al. Colon cancer mesenchymal stem cells modulate the tumorigenicity of colon cancer through interleukin 6. Exp Cell Res. 2013;319(14):2216–29. 10.1016/j.yexcr.2013.06.003. 23751564

[45] Chung YC, Chang YF. Serum interleukin-6 levels reflect the disease status of colorectal cancer. J Surg Oncol. 2003;83(4):222–6. 10.1002/jso.10269. 12884234

[46] Xu J, Ye Y, Zhang H, Szmitkowski M, Makinen MJ, Li P, et al. Diagnostic and prognostic value of serum interleukin-6 in colorectal cancer. Med (Baltim). 2016;95(2):e2502. 10.1097/MD.0000000000002502. PMC4718291 26765465

[47] Knupfer H, Preiss R. Serum interleukin-6 levels in colorectal cancer patients--a summary of published results. Int J Colorectal Dis. 2010;25(2):135–40. 10.1007/s00384-009-0818-8. 19898853

[48] Yeh KY, Li YY, Hsieh LL, Lu CH, Chou WC, Liaw CC, et al. Analysis of the effect of serum interleukin-6 (IL-6) and soluble IL-6 receptor levels on survival of patients with colorectal cancer. Jpn J Clin Oncol. 2010;40(6):580–7. 10.1093/jjco/hyq010. 20194250

[49] Shiga K, Hara M, Nagasaki T, Sato T, Takahashi H, Sato M, et al. Preoperative serum interleukin-6 is a potential prognostic factor for colorectal cancer, including stage II patients. Gastroenterol Res Pract. 2016;2016:9701574. 10.1155/2016/9701574. PMC4706938 26858756

[50] Sasaki Y, Takahashi T, Miyazaki H, Matsumoto A, Kato T, Nakamura K, et al. Production of thrombopoietin by human carcinomas and its novel isoforms. Blood. 1999;94(6):1952–60. 10.1182/blood.V94.6.1952. 10477724

[51] Dymicka-Piekarska V, Kemona H. Thrombopoietin and reticulated platelets as thrombopoietic markers in colorectal cancer. Thromb Res. 2008;122(1):141–3. 10.1016/j.thromres.2007.10.003. 18061247

[52] Zhou CL, Su HL, Dai HW. Thrombopoietin is associated with a prognosis of gastric adenocarcinoma. Rev Assoc Med Bras (1992). 2020;66(5):590–5. 10.1590/1806-9282.66.5.590. 32638965

[53] Singh S, Earle CC, Bae SJ, Fischer HD, Yun L, Austin PC, et al. Incidence of diabetes in colorectal cancer survivors. J Natl Cancer Inst. 2016;108(6):djv402. 10.1093/jnci/djv402. 26839345

[54] Peeters PJ, Bazelier MT, Leufkens HG, de Vries F, De Bruin ML. The risk of colorectal cancer in patients with type 2 diabetes: associations with treatment stage and obesity. Diabetes Care. 2015;38(3):495–502. 10.2337/dc14-1175. 25552419

[55] Overbeek JA, Kuiper JG, van der Heijden A, Labots M, Haug U, Herings RMC, et al. Sex- and site-specific differences in colorectal cancer risk among people with type 2 diabetes. Int J Colorectal Dis. 2019;34(2):269–76. 10.1007/s00384-018-3191-7. PMC6331739 30421309

[56] Zhu B, Wu X, Wu B, Pei D, Zhang L, Wei L. The relationship between diabetes and colorectal cancer prognosis: a meta-analysis based on the cohort studies. PLoS One. 2017;12(4):e0176068. 10.1371/journal.pone.0176068. PMC5397066 28423026

[57] Petrelli F, Ghidini M, Rausa E, Ghidini A, Cabiddu M, Borgonovo K, et al. Survival of colorectal cancer patients with diabetes mellitus: a meta-analysis. Can J Diabetes. 2021;45(2):186–97. e2. 10.1016/j.jcjd.2020.06.009. 33039329

[58] Kraakman MJ, Lee MK, Al-Sharea A, Dragoljevic D, Barrett TJ, Montenont E, et al. Neutrophil-derived S100 calcium-binding proteins A8/A9 promote reticulated thrombocytosis and atherogenesis in diabetes. J Clin Invest. 2017;127(6):2133–47. 10.1172/JCI92450. PMC5451242 28504650

[59] Liu C, Feng X, Li Q, Wang Y, Li Q, Hua M. Adiponectin, TNF-alpha and inflammatory cytokines and risk of type 2 diabetes: A systematic review and meta-analysis. Cytokine. 2016;86:100–9. 10.1016/j.cyto.2016.06.028. 27498215

[60] Pletsch-Borba L, Watzinger C, Turzanski Fortner R, Katzke V, Schwingshackl L, Sowah SA, et al. Biomarkers of vascular injury and type 2 diabetes: a prospective study, systematic review and meta-analysis. J Clin Med. 2019;8(12):2075. 10.3390/jcm8122075. PMC6947572 31783601

[61] Santilli F, Pignatelli P, Violi F, Davi G. Aspirin for primary prevention in diabetes mellitus: from the calculation of cardiovascular risk and risk/benefit profile to personalised treatment. Thromb Haemost. 2015;114(5):876–82. 10.1160/TH15-03-0202. 26245672

[62] American Diabetes Association. 10. Cardiovascular disease and risk management: standards of medical care in diabetes-2020. Diabetes Care. 2020;43(S1):S111–34. 10.2337/dc20-S010. 31862753

